# Hypoxia differentially regulated CXCR4 and CXCR7 signaling in colon cancer

**DOI:** 10.1186/1476-4598-13-58

**Published:** 2014-03-14

**Authors:** Benoît Romain, Muriel Hachet-Haas, Serge Rohr, Cécile Brigand, Jean-Luc Galzi, Marie-Pierre Gaub, Erwan Pencreach, Dominique Guenot

**Affiliations:** 1 Service de Chirurgie Générale et Digestive, Hôpitaux Universitaires de Strasbourg, Hôpital de Hautepierre, Avenue Molière, 67098, Cedex, Strasbourg, France; 2 Université de Strasbourg (UdS), EA 3430 Progression tumorale et microenvironnement. Approches translationnelles et Epidémiologie. Fédération de Médecine Translationnelle de Strasbourg (FMTS), Bâtiment U1113, 3 Avenue Molière, 67200 Strasbourg, France; 3 Ecole supérieure de biotechnologie (ESBS), CNRS/UdS UMR 7242 and Laboratory of Excellence « Medalis », Parc d'innovation Boulevard Sébastien Brant, BP 10413, 67412, Cedex, Illkirch, France; 4 Laboratoire de Biochimie et Biologie Moléculaire, Hôpitaux Universitaires de Strasbourg, Hôpital de Hautepierre, Avenue Molière, 67098 Cedex, Strasbourg, France; 5 Centre de Ressources Biologiques, Hôpitaux Universitaires de Strasbourg, Strasbourg, France

**Keywords:** Chemokines, Hypoxia, Colon, Migration, Metastasis

## Abstract

**Background:**

HIF-1α and CXCR4/CXCL12 have crucial roles in the metastatic process of colorectal cancer. Our aim was to study the significance of targeting HIF-1α and the CXCR4/CXCL12 axis in colorectal cancer to prevent the dissemination process *in vitro*.

**Methods:**

We investigated CXCR4 and CXCR7 mRNA and protein expression in human colon carcinomas and the modulation of their expression by hypoxia and HIF-1α in colon cancer cell lines. The migration of tumor cells in a Boyden chamber was studied after CXCR4 inhibition with siRNA or the CXCR4/CXCL12 neutraligand, chalcone 4.

**Results:**

Analysis of a cohort of colon polyps and chromosome-unstable carcinomas showed that the expression of CXCR4 and CXCR7 was similar to that of the normal mucosa in the polyps and early-stage carcinomas but significantly increased in late stage carcinomas. Our data demonstrate that hypoxia strongly induced the expression of CXCR4 transcript and protein at the cell membrane, both regulated by HIF-1α, whereas CXCR7 expression was independent of hypoxia. After transient hypoxia, CXCR4 levels remained stable at the cell membrane up to 48 hours. Furthermore, reducing CXCR4 expression impaired CXCL12-induced Akt phosphorylation, whereas Erk activation remained unchanged. In contrast, reducing CXCR7 expression did not affect Akt nor Erk activation. In the presence of CXCR4 or CXCR7 siRNAs, a significant reduction in cell migration occurred (37% and 17%, respectively). Although irinotecan inhibited cell migration by 20% (p <0.001), the irinotecan and chalcone 4 combination further increased inhibition to 40% (p <0.001).

**Conclusion:**

We demonstrated, for the first time, that hypoxia upregulated CXCR4 but not CXCR7 expression in tumor cells and that the CXCR4 receptor protein level remains high at the cell membrane when the tumor cells return to normoxia for up to 48 hours. In addition we showed the interest to inhibit the CXCR4 signaling by inhibiting both the HIF-1α and CXCR4/CXCL12 pathway. CXCR4 seems to be a relevant target because it is continuously expressed and functional both in normoxic and hypoxic conditions in tumor cells.

## Introduction

Tumor progression is associated with intratumoral hypoxia, which leads to an increase in vascular density. The increased vascular density often exhibits an abnormal architecture and provides heterogeneous perfusion within the tumor tissue [1]. HIF-1α is a transcription factor that permits the adaptation of tumor cells to changing environment, such as hypoxia [2]. Many studies have shown that HIF-1α is overexpressed at very high levels in colorectal tumors, particularly in the most aggressive tumors [3]. HIF-1α protein plays a major role in regulating the expression of many genes involved in angiogenesis and erythropoiesis, metabolic adaptation to hypoxia, epithelial-mesenchymal transition (EMT), extracellular matrix degradation and chemotaxis through CXCR4 and the CXCL12/SDF-1 axis [4].

The expression of chemo-attractant molecules and their receptors (such as CXCL12-SDF1/CXCR4 and VEGF/VEGFRs) induces tumor cell dissemination from primitive tumor sites to metastatic niches. In many tumor models, these molecules permit tumor cell survival in the metastatic microenvironment and the recruitment of hematopoietic and endothelial progenitors for neovascularization [5]. Interestingly, recent studies have shown that overexpressions of the chemokine receptor CXCR4 and of VEGF were predictive of early distant relapse in stages II and III colorectal cancers [6]. CXCR4 is a highly conserved G protein coupled receptor (GPCR) that binds CXCL12. Although CXCR4 is expressed in a wide range of tissues, its expression is low or absent in normal tissues and becomes important in malignant cells of many human cancers types, including breast cancer, ovarian cancer, melanoma, prostate cancer and colorectal cancer [7]. Its ligand, CXCL12, is constitutively and physiologically expressed in the liver, lungs, lymph nodes and bone marrow [8,9]. CXCR7 is another GPCR which also binds to CXCL12, but with a ten fold greater affinity compared to CXCR4 [10]. Although the role of CXCL12/CXCR7 signaling is not yet fully described, this receptor seems to be essential for the survival and growth of tumor cells [11-14].

Due to the crucial role of HIF-1α and CXCR4/CXCL12 in the metastatic process of colorectal cancer, we determined *CXCR4* and *CXCR7* gene expression in human colon carcinomas and their modulation by hypoxia and HIF-1α in colon cancer cell lines. We found that the CXCR4 and CXCR7 expression levels increased proportionally to the clinical stage and that hypoxia differentially regulated the receptors. Furthermore, CXCR4 remained stably expressed at the cell membrane after transient hypoxia followed by 24 or 48 hours of normoxia. We also found that inhibition of CXCR4 with siRNA or with the CXCR4/CXCL12 neutraligand chalcone 4 significantly decreased the migration of these cells *in vitro*, an effect that was amplified by concomitant inhibition of HIF-1α. Taken together, these results indicate the potential of targeting HIF-1α and CXCR4/CXCL12 in colorectal cancer.

## Results

### *In vivo* CXCR4 and CXCR7 expression in human carcinomas

**Figure 1:**
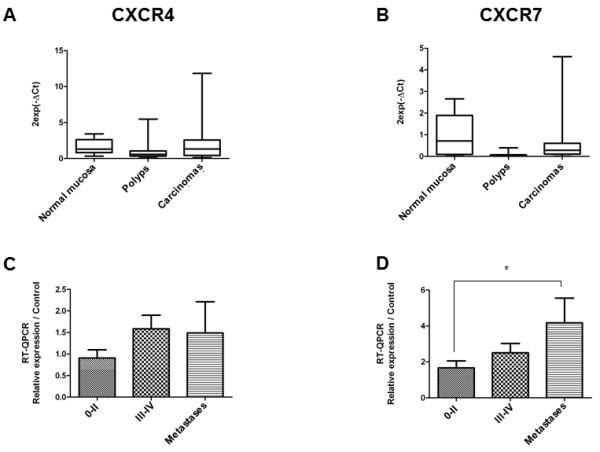
**CXCR4 and CXCR7 expression. A**. CXCR4 expression in normal mucosa, polyps and carcinomas; **B**. CXCR4 expression according to UICC stages. **C**. CXCR7 expression in normal mucosa (n=), polyps (n = 30) and carcinomas (n = 46); **D**. CXCR7 expression according to UICC stages. *p = 0.02.

**Figure 2:**
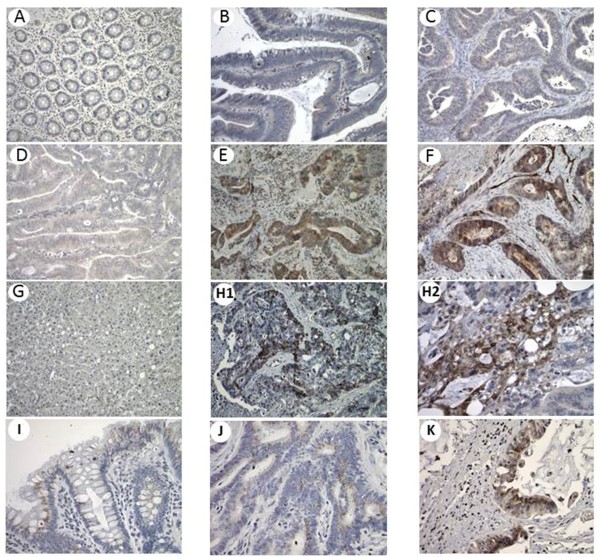
**CXCR4 and CXCR7 protein expression in human colon polyps, early and late stage carcinomas and metastases.** Detection of CXCR4 protein expression assessed by immunohistochemical staining in representative specimens of polyp **(B)**, primary stage I **(C)**, II **(D)**, III **(E)**, IV **(F)** colon tumours, metastatic liver **(H1&H2)** and corresponding non-cancerous neighbour colon **(A)**, and liver **(G)** tissues. Detection of CXCR7 protein expression in non-cancerous neighbour colon **(I)**, primary stage IV colon tumor **(J)** and corresponding metastatic liver **(K)**. Original magnification x200 (except H2, x400).

We measured CXCR4 and CXCR7 mRNA levels in human polyps (n = 30) and carcinomas (n = 46). Tumor stages were classified according to UICC (International Union Against Cancer) recommendations (Table 1). The expression of CXCR4 and CXCR7 mRNA in these tumors was compared to that of a pool of healthy colonic mucosa samples. Overall, the expression levels of both CXCR4 and CXCR7 were identical to those of the normal mucosa (Figure 1A-B). In carcinomas, CXCR4 expression significantly increased (p = 0.035) between early stage tumors (0-II) and late stage tumors (III-IV) but without significance between stages III-IV and metastases (p = 0.45) Figure 1C). CXCR7 expression significantly increased between early stage tumors (0-II) and metastases (Figure 1D) (p = 0.02). Similarly, the CXCR4 and CXCR7 protein expression was absent in polyps and early stages carcinomas but increased between stage II and III, to be highest in the stage IV carcinomas (Figure 2A-G). Expression was maintained in the liver metastases (Figure 2H). Concerning CXCR7, a weak expression was found in the normal mucosa and stages I to IV, which increased in the liver metastases due to increased number of cells expressing CXCR7 (Figure 2I-K).

**Table 1:** Characteristics of adenomas and carcinomas

**Adenomas**			**Carcinomas**		
**Mean Age (years)**		**66.5 ± 10.2**	**Mean Age (years)**		**66.1 ± 12**
		**Number (n)**			**Number (n)**
**Total Adenomas**		**30**	**Total Carcinomas**		**46**
**Men :Women**		15 :11	**Men :Women**		33 :13
**Localisation**	**Proximal colon**	14	**Localisation**	**Proximal colon**	19
	**Distal colon**	12		**Distal colon**	22
				**Metastasis**	5
**UICC classification**	**Villous**	1	**UICC classification**	**0 (Tis)**	2
	**Hyperplasia**	0		**I**	7
	**Tubulovillous**	2		**II**	12
	**Tubular**	23		**III**	10
**Grade**	**High**	6		**IV**	15
	**Low**	20			

### *In vitro* CXCR4 expression is regulated by HIF-1α

#### ***Transcript expression***

**Figure 3:**
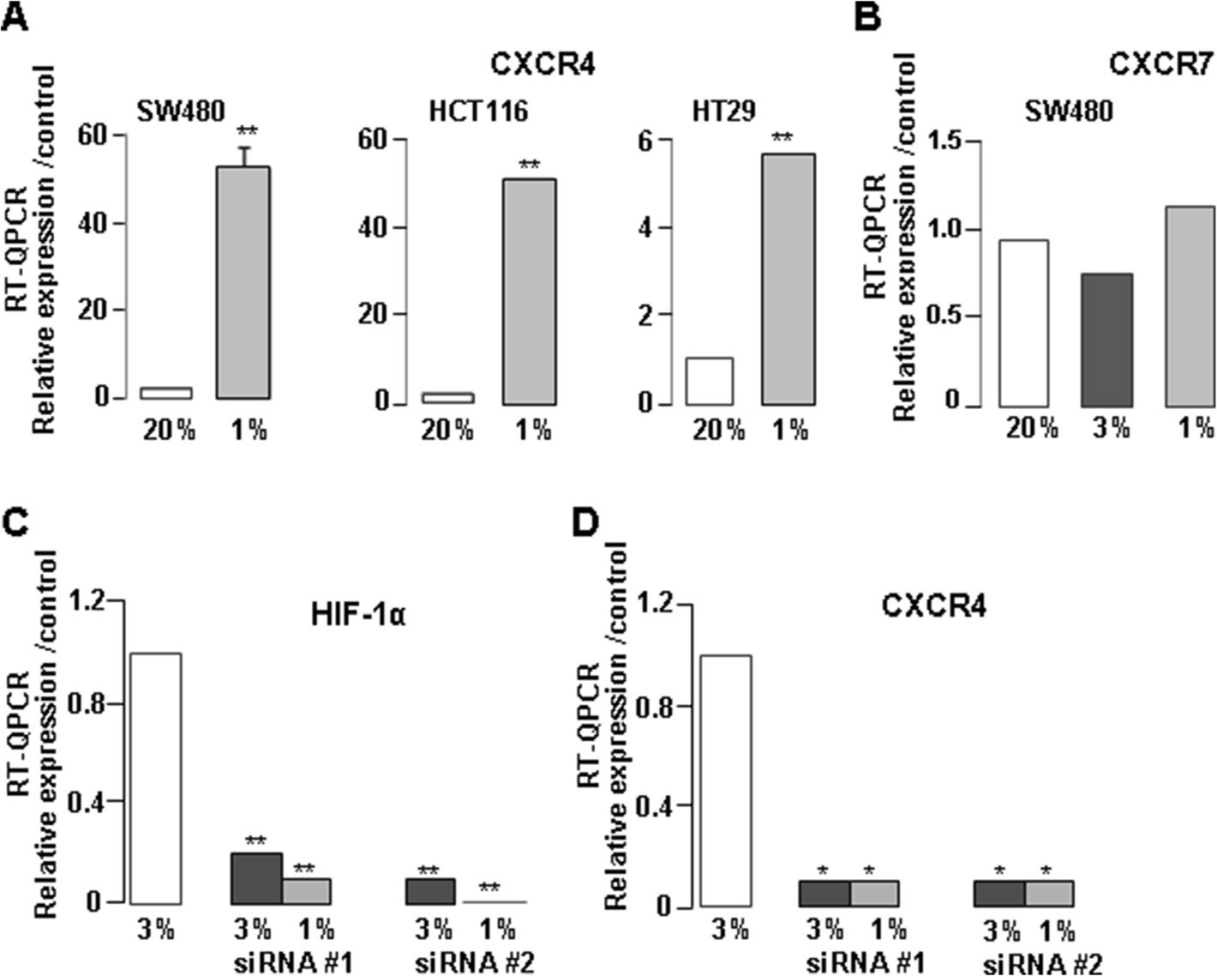
**CXCR4 expression in different colon cell lines cultured in hypoxia. (A)** Transcript CXCR4 expression in normoxia (20% O2) and hypoxia (1% O2) in SW480, HCT116 and HT29 cell lines. **(B)** Transcript CXCR7 expression in normoxia (20% O2) and in hypoxia (3% and 1% O2) in SW480 cell line. Trancript HIF-1α **(C)** and CXCR4 **(D)** expression after siRNA #1 or #2 anti-HIF-1α transfection; * p = 0.015; ** p = 0.001.

**Figure 4:**
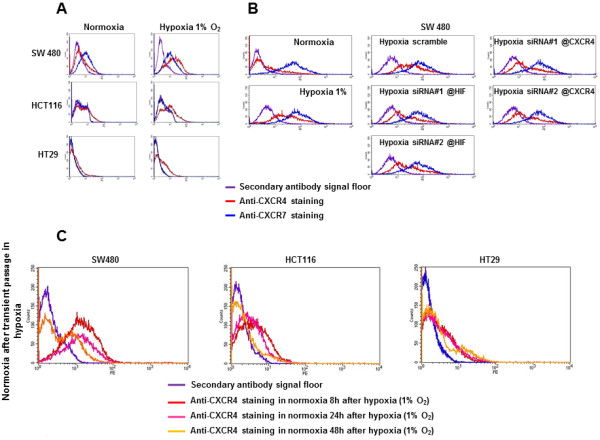
**Regulation of CXCR4 and CXCR7 protein expression at the cell membrane using flow cytometry. (A)** CXCR4 and CXCR7 expression at the cell membrane of SW480, HCT116, and HT29, in normoxia (20% O2) and hypoxia (1% O_2_). **(B)** CXCR4 and CXCR7 expression in SW480 cells after siRNA anti-HIF-1α or anti-CXCR4 in hypoxia. **(C)** Cell membrane CXCR4 protein expression after transient passage in hypoxia. SW480, HCT116 and HT29 cells were cultured 24 hours in 3% hypoxia and further maintained for 8, 24 and 48 hours in normoxia.

We studied CXCR4 mRNA expression in normoxia (20% O_2_) and hypoxia (3% or 1% O_2_) in SW480, HCT116 and HT29 cells. In hypoxia, there was a significant increase in CXCR4 mRNA level for the three cell lines (50 fold, 5.5 fold and 52.7 fold for SW480, HT29 and HCT116 cells respectively) (Figure 3A) with a higher increase at 1% O_2_ than at 3% O_2_ (data not shown). On the other hand, as compared to SW480 cells, HT29 and HCT116 cell lines express low rates of CXCR7 (HT29 < HCT116 < SW480, see Figure 4), and in SW480 cells, no change in CXCR7 mRNA level was observed between the normoxic and hypoxic conditions (Figure 3B). To determine if the hypoxia-related increase of CXCR4 mRNA expression is regulated by HIF-1α, we used two different HIF-1α siRNAs. HIF-1α mRNA expression was inhibited by 90% with both HIF-1α siRNAs (Figure 3C), and CXCR4 expression was concomitantly inhibited by more than 90% (Figure 3D).

#### ***Protein expression***

We then studied the regulation of CXCR4 and CXCR7 protein expression at the cell membrane in hypoxia using flow cytometry. For the three cell lines, hypoxia (1% 02) upregulated the expression of CXCR4 protein at the cell membrane, whereas CXCR7 expression remained unchanged (Figure 4A), confirming the transcriptional data. The HIF-1α and CXCR4 siRNAs led to decreased CXCR4 protein expression, but not to the basal level observed in normoxia (Figure 4B). These data demonstrate that hypoxia induces a strong expression of CXCR4 at the cell membrane that is regulated by HIF-1α, whereas CXCR7 expression is independent of hypoxia, HIF-1α and CXCR4. Flow cytometry confirmed our previous observation that CXCR7 is absent in the HCT116 and HT29 cells but highly expressed in the SW480 cells.

#### ***Cell membrane CXCR4 expression after transient passage in hypoxia***

Within the tumor microenvironment, cells are subjected to cycles of hypoxia/normoxia [15], whereas in the bloodstream, they are exposed to normoxia. We hypothesized that even after a transient passage through hypoxia, circulating tumor cells, although exposed to normoxic conditions in the bloodstream, will maintain CXCR4 expression at the cell membrane, allowing the metastatic process via the CXCL12 gradient between the primary tumor site and the liver. Flow cytometry allowed us to analyze CXCR4 expression in the colon cell lines cultured 24 hours in hypoxia (1% O_2_) and further maintained for 8, 24 and 48 hours in normoxia. CXCR4 protein level remained elevated at the cell membrane in the three cell lines for 8 to 24 hours. At 48 hours CXCR4 immunoreactivity remained significantly higher in HT29 and SW480 cells (Figure 4C).

### Akt and Erk oncogenic pathways are strongly activated by stimulation of the CXCR4/CXCL12 axis *in vitro*

#### ***Modulation by CXCL12***

**Figure 5:**
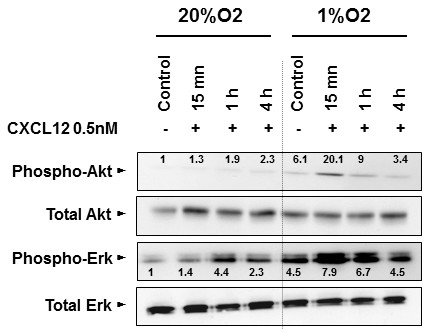
**Activation of Akt and Erk oncogenic pathways by stimulation of CXCR4/CXCL12 axis in SW480 cell line.** Effect of CXCL12 stimulation on Akt and Erk oncogenic pathways. SW480 cells were stimulated with CXCL12 at concentration of 0.5 nM. The cells cultivated either in normoxia or in hypoxia (1%O2) and previously starved for 4 h in a serum-free medium before adding CXCL12, were evaluated for protein kinase phosphorylation.

Our aim was to analyze the effect of CXCL12 stimulation on the Akt and Erk oncogenic pathways. SW480 cells were stimulated with 0.5 nM and 50 nM CXCL12. The cells were starved for 4 h in a serum-free medium before adding CXCL12, then maintained either in normoxia or in hypoxia (1%O_2_) and evaluated for protein kinase phosphorylation. In normoxia, both pathways were activated with CXCL12 (Figure 5). This activation, however, was weak as compared to that observed in hypoxia at 15 minutes, more specifically for the level of Akt phosphorylation (Figure 5). Increasing the CXCL12 concentration to 50 nM did not further enhance the level of Akt and Erk phosphorylation (data not shown).

#### ***Impact of CXCR4/CXCL12 axis inhibition***

To study the effect of CXCR4/CXCL12 axis inhibition on Akt and Erk phosphorylation, the SW480 cell line was treated with CXCR4 and CXCR7 siRNAs in hypoxia (1% O_2_) in the presence of 0.5 nM CXCL12 for 15 minutes. Reducing CXCR4 expression impaired CXCL12-induced Akt phosphorylation, whereas Erk activation remained unchanged.

**Figure 6:**
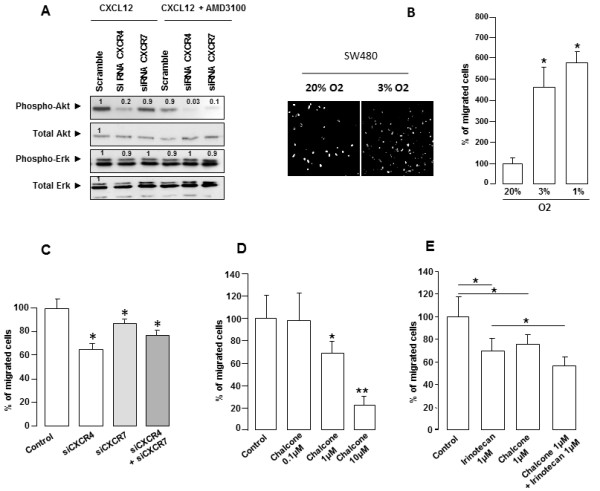
**Impact of CXCR4/CXCL12 axis inhibition in SW480 cell line. (A)** Effect of CXCR4/CXCL12 axis inhibition on Akt and Erk phosphorylation. Cells are treated with CXCR4 and CXCR7 siRNAs in hypoxia (1% O_2_) in presence of 0.5 nM CXCL12 for 15 minutes with or without the CXCR4 antagonist AMD 3100 (10 μM). **(B)** SW480 cell migration in hypoxia (3% and 1% O2) using a Boyden chamber assay. After 24 h incubation, cells remaining on the upper chamber are mechanically removed. The cells that have migrated to the lower chamber are counted after staining with fluorescent dye (DAPI or 4',6-diamidino-2-phenylindole). Quantification was performed by microscope counting five random fields for each chamber (* p < 0.001). **(C)** SW480 cell migration was analyzed in presence of CXCR4 or CXCR7 siRNA. There was a significant reduction in migration (37% and 17%, respectively. **(D)** The effect of increasing doses of chalcone 4 (0.1 μM, 1 μM and 10 μM) was measured on cells migrating under hypoxic conditions. Chalcone 4 at 1 μM and 10 μM reduced migration by 23% and 80%, respectively. **(E)** Effect of the combination of chalcone 4 and irinotecan at 1 μM. Although irinotecan inhibited migration by 20% (*p <0.01), the irinotecan and chalcone 4 combination further increased the inhibition to 40% (**, p = 0.001).

Simultaneously blocking the CXCL12-CXCR4 interaction with AMD3100 and inhibiting CXCR4 expression with siRNA fully blocked CXCL12-induced Akt activation, still without affecting Erk activation. Targeting CXCR7 and CXCL12/CXCR7 interaction affected neither Akt nor Erk signaling (Figure 6A). Thus, the CXCL12/CXCR4 axis, but not the CXCL12/CXCR7 axis, is able to modulate the Akt pathway.

#### ***Therapeutic significance of targeting HIF-1α and CXCR4/CXCL12 to prevent the dissemination process in vitro***

The migration of SW480 cells was significantly increased in hypoxia (4.6 fold at 3% and 5.8 fold at 1% O2) compared to normoxia (p < 0.01) (Figure 6B). In the presence of CXCR4 or CXCR7 siRNA, a significant reduction in cell migration occurred (37% (p < 0.01) and 17%, respectively (p < 0.01)) (Figure 6C). Treatment with both CXCR4 and CXCR7 siRNAs did not further decrease cell migration (25.5% inhibition; p < 0.01).

Previous studies showed that irinotecan, a standard chemotherapeutic drug for metastatic colon cancer, has a cytostatic effect on xenografted colon tumors through the inhibition of HIF-1α expression [16]. We also previously identified the compound chalcone 4 as a neutraligand against CXCR4/CXCL12 interaction that blocks CD4^+^ peripheral blood lymphocytes migration towards CXCL12 [17]. Therefore, we decided to test the combined effects of irinotecan and chalcone 4 on tumor cell migration. First, the effect of increasing doses of chalcone 4 (0.1 μM, 1 μM and 10 μM) was measured on SW480 cells migration under hypoxic conditions. Chalcone 4 at 1 μM and 10 μM reduced migration by 23% and 80% (p <0.01), respectively (Figure 6D). Based on these data, we used the combination of 1 μM chalcone 4 and 1 μM irinotecan (Figure 6E). These results clearly show that chalcone 4 or irinotecan reduces cell migration by 20% at 1 μM. Upon co-treatment the inhibition is further increased this inhibition to 40% (p <0.001). These results thus demonstrate the significance of inhibiting HIF-1α and the CXCR4/CXCL12 interaction to affect the process of *in vitro* cell dissemination.

## Discussion

Analysis of a cohort of colon polyps and chromosome-unstable carcinomas showed that the expression of CXCR4 and CXCR7 was similar to that of the normal mucosa in the early-stage but significantly increased from early to late stage carcinomas. Using three colon cell lines, we showed that hypoxia was a strong activator of CXCR4 expression, mainly through the involvement of HIF-1α, whereas CXCR7, only expressed in SW480 cells, was not modulated by hypoxia or HIF-1α. In addition, we showed for the first time that after transient passage in hypoxia, CXCR4 remained expressed at the cell membrane when exposed to normoxia for up to 48 hours. Finally, a novel combination of an HIF-1α inhibitor (irinotecan) and a CXCL12-CXCR4 interaction inhibitor (chalcone 4) significantly impaired the *in vitro* cell migration process. Although the migration inhibition is only partial (40%), the fact that a higher chalcone concentration (10 μM) inhibits migration by 80%, is clearly in favor of the involvement of CXCL12 via CXCR4 in the tumor cell migration process.

Recent studies have reported on the overexpression of the chemokine receptors CXCR4 and CXCR7 by several tumor entities and have shown that CXCR4 plays a crucial role in organ-specific metastasis formation [18]. However, the precise mechanisms of chemokine receptor-driven homing of cancer cells to specific sites of metastasis remain unclear.

Angiogenesis is critical to the growth, invasion, and metastasis of human tumors [19,20]. Because targeting angiogenesis has emerged as a promising strategy for the therapeutic treatment of cancer, understanding the molecular mechanism linking tumor angiogenesis to the potential of a tumor to disseminate has become very important. Dysregulation of HIF and/or cytokines, such as the CXCR4/CXCR7/CXCL12 axis, is one probable cause of increased angiogenesis via the overexpression of tumor VEGF. This has led to the development of targeted therapies such as an anti-VEGF antibody, recently approved for clinical use [21]. However, other mechanisms are most likely responsible for tumor progression and dissemination, and the interaction between CXCL12 and its receptor CXCR4 was shown to play a major role in the settlement of colorectal tumor cells in the liver [22]. Although CXCR4 expression is low or absent in normal tissues, CXCR4 is overexpressed in many cancer types, including melanoma, breast, ovarian, prostate and colorectal cancers [7,18]. In contrast, the chemokine CXCL12, expressed at the surface of normal intestinal epithelium [23], is decreased in tumor tissues, such as colon or breast carcinomas [24,25]. As previously shown in glioblastoma cells, hypoxia and HIF-1α can regulate the expression of CXCR4 in colon cancer cell lines [26]. Others have shown that hypoxia increases CXCR4 expression through HIF-1α activation and that HIF-1α enhances the expression and function of CXCR4 in normal cells monocytes, macrophages and endothelial cells [27] and in tumor cells [28]. In our hands, siRNAs targeting HIF-1α prevented both HIF-1α and CXCR4 upregulation under hypoxic conditions.

In human colon carcinomas, we observed that CXCR4 expression significantly increased during tumor progression as it increased from stages 0-II to III-IV, whereas for CXCR7, a significant increase was observed between early stages and liver metastases. Knowing that metastases develop from circulating tumor cells escaping the primary site of cancer during their passage in the blood stream [29], these cells switch from a hypoxic to a normoxic environment and escape regulation by HIF-1α. Fitting with this hypothesis, we demonstrated for the first time that after a transient passage through hypoxia, which leads to the upregulation of CXCR4 expression, the receptor protein level remains high at the cell membrane even when the cells returned back to normoxia. The maintenance of high CXCR4 level could help circulating cells to home in organs expressing high levels of the CXCL12 ligand, and with the resident CXCR7 may aid endothelial extravasation favoring metastasis development [30,31]. During embryogenesis, it has been shown that CXCR7 is only expressed in the trailing cells of the primordium and is required to provide migration directionality [32].

The CXCR4/CXCL12 interaction provokes calcium mobilization and activation of multiple signaling pathways, including PI3K/Akt, PLC-γ/Protein kinase C and Erk/Ras [33,34]. We show that hypoxia alone rapidly activated the PI3K/Akt and Erk/Ras pathways and that this effect was amplified under short-term CXCL12 stimulation. Interestingly, part of the PI3K/Akt activation was induced by the interaction of CXCL12 with CXCR4, as it was blocked by siRNA targeting CXCR4, but not by its interaction with CXCR7. As PI3K/Akt activation could not be totally abolished with siRNA targeting CXCR4, other receptors may participate in the activation of this oncogenic pathway, although at present no other receptors have been shown to interact with CXCL12. Nevertheless, the short-term activation of the oncogenic pathway may be sufficient to initiate the migration process observed when cells are switched to hypoxia, and this activation could be blocked with a siRNA against CXCR4.

Although CXCR4 inhibition with siRNA or AMD3100 affected the PI3K/Akt pathway, no change in activity was observed for the Erk/Ras pathway. A number of the components of this PI3K/Akt pathway are mutated or deregulated in a wide variety of human tumors, highlighting the key role of this pathway in cellular transformation [35]. Following Akt phosphorylation, the subsequent phosphorylation of its targets regulates a variety of critical cell functions, including glucose metabolism, cell proliferation and survival. PI3K also is likely implicated in the metastatic phenotype. Indeed, several molecules involved in cell migration and cell adhesion can regulate -or be regulated by- PI3K. Indeed, PI3K/Akt was shown to be essential for Matrix Metalloproteinase (MMP) production in several cell lines [36] and clinical and animal studies revealed that PI3K/Akt activates MMP-2, MMP-9, and Urokinase-type plasminogen activator (uPA), leading to destruction of the extracellular matrix [37]. Other data might explain our observation of inhibition of cell migration with CXCR4 inhibitors. Gassmann and colleagues for instance, demonstrated that colon cell line extravasation into the liver parenchyma is regulated *in vivo* by CXCL12-activated CXCR4 [30]. In contrast, we found that CXCR7 silencing did not modify the migration process or the activation of the PI3K/Akt or Erk/Ras pathway. This is consistent with recent studies providing alternative mechanisms through which CXCR7 can regulate CXCL12-directed cell movement. CXCR7 does not appear to induce cell migration directly but may enhance cell adhesion [11], and the involvement of CXCR7 in CXCL12-mediated transendothelial migration of human renal multipotent progenitor cells has been demonstrated [12]. Additionally, Zabel et al showed that CXCR7, through association with β-arrestin but without Ca^2+^ mobilization, regulates the ability of human CXCR7^+^/CXCR4^+^ lymphoblastoid cells to migrate across an endothelial cell monolayer [38].

HIF-1α is frequently upregulated at protein level in response to the hypoxic tumor environment and this overexpression has been associated with an aggressive phenotype, namely resistance to chemotherapy and poor outcome in a wide range of tumors [3]. One hypothesis concerning the metastatic process is based on an increasing CXCL12 gradient from the primary tumor to secondary niches at metastatic sites. Immunohistochemical analyses have shown that CXCL12 is highly expressed in hepatic sinusoids including endothelial and Kupffer cells [38] and that disseminating tumor cells express CXCR4 [7]. Thus, the CXCL12/CXCR4 interaction permits extravasation of colon tumor cells in the liver parenchyma [36]. Moreover, CXCR4/CXCL12 interaction increases the expression of proteins important for cell migration, motility and invasion, such as Rho and Rac [39-41].

Altogether, our results demonstrate the potential value of inhibiting HIF-1α and CXCR4/CXCL12 to counteract the migration process. We have used an innovative approach to impair tumor cell migration by combining irinotecan and chalcone 4 that could be of therapeutic interest. We have previously shown that irinotecan inhibited HIF-1α protein accumulation in *in vitro*[42] and *in vivo* models of colon cancer [16,42]. We hypothesized that irinotecan would inhibit CXCR4 expression by inhibiting HIF-1α. Chalcone 4 is a neutraligand of CXCL12 and impairs CXCR4/CXCR7/CXCL12 interaction. In addition, other studies have already shown that inhibition of CXCR4 *in vivo* inhibits the metastatic process and the migration of breast cancer cells. We have shown that the combination of the two drugs is more effective than each drug separately as migration was decreased by more than 40%.

## Conclusion

We have demonstrated for the first time the potential therapeutic significance of inhibiting CXCR4 signaling through a combinatorial approach inhibiting HIF-1α and CXCR4/CXCL12 interaction. CXCR4 seems to be a relevant target, as CXCR4 remains continuously expressed when tumor cells switch from a hypoxic to a normoxic environment. Finally, CXCR7 is differentially expressed compared to CXCR4 and could be involved in some subtypes of more aggressive tumors. Thus, CXCR4 and CXCR7 seem to play different roles in colon tumors, and further studies are necessary to better understand their respective roles.

## Materials and methods

### Tumor specimens

Human tumor specimens were obtained at the Gastrointestinal Surgical Department of the University Hospital Hautepierre (Strasbourg-France) according to the French Ethical Committee recommendations and the ethical standards of the 1964 Declaration of Helsinki. All patients provided written informed consent.

### Cell culture and treatments

Human colon carcinoma HCT-116, HT-29 and SW480 cells were maintained at 37°C under normoxic (20% O_2_) and hypoxic conditions (94% N_2_, 5% CO_2_, 3 or 1% O_2_, Sanyo) in DMEM (1 g/L glucose) supplemented with 10% fetal bovine serum. The cells were treated during exponential growth conditions (30% confluence).

Irinotecan (Campto^®^, irinotecan chlorydrate, Pfizer) was used at a concentration of 1 μM. AMD3100 (Sigma France), an antagonist of CXCR4, was used at 10 μM. Chalcone 4 was provided by JL Galzi (ESBS, Strasbourg, France); [17].

### SiRNA transfections

The cells were transfected in 6-well plates with siRNA anti-HIF-1α (20 nM; siRNA 1: Hs_HIF-1α_5: SI02664053, siRNA 2: Hs_ HIF-1α_6: SI02664431, Qiagen^®^) and anti-CXCR4 (20 nM; siRNA 1: Hs_CXCR4 7: SI02664235 and siRNA 2: Hs_CXCR4 8: SI02664242, Qiagen^®^) with the Lipofectamine^®^ RNAiMAX, (Invitrogen^®^, Life Technologies) according to the manufacturer’s instructions. A non-specific, non-targeting siRNA was used as the control treatment (Eurogentec^®^). The cells were incubated for 48 h at 37°C in hypoxia (94% N_2_, 5% CO_2_, 3 or 1% O_2_, Sanyo^®^).

### Migration tests

Boyden chambers (BD Biosciences) were used for the *in vitro* migration assay. The upper and lower compartments were filled with 1% FCS and 10% FCS, respectively. Cells (5 × 10^5^) were added in the upper compartment. After 24 h, the cells were fixed with 4% paraformaldehyde for 15 min and stained with DAPI (1/30000; 4', 6'-diamidino-2-phenyl indole, Sigma^®^). Migrating cells were counted using an epifluorescence microscope.

### Relative quantitative PCR

The mRNA expression of CXCR4, CXCR7, HIF-1α and control PDGF genes was evaluated by relative quantitative real-time PCR (RT-qPCR) analysis using the LightCycler system (Roche Molecular Biochemicals^®^) and FastStart DNA Master Mix SYBR Green I (Roche Diagnostics^®^). RNA was extracted with Trizol reagent (Invitrogen^®^) according to the manufacturer's protocol. Reverse transcription of 2 μg RNA was performed using reverse transcriptase and oligo(dT) primers (FinnZyme^®^). PCR was performed as follows: denaturation at 95°C for 10 min, followed by 40 cycles at 95°C for 20 s and 62°C for 20 s and elongation at 72°C for 20 s using the maximum temperature transition rate of 20°C/s. Fluorescence measurements were taken at the end of the elongation phase. The specificity of the PCR products was assessed by generating a melting curve. All quantifications were performed in duplicate for three independent experiments and normalized with respect to the endogenous PDGF mRNA levels. Target cDNA expression was quantified using the ΔΔCt method. Validated primers were obtained from Qiagen^®^.

### Western Blot

Western Blots were performed with the following antibodies: anti-Akt total (1/1000), anti-Erk total (1/2000), anti-phospho Akt (1/1000), anti-phospho-Erk (1/1000) (from Cell Signaling Technology ^®^), anti-HIF-1alpha (1/1500, BD Biosciences^®^), anti-actin (1/15000, Millipore ^®^).

### Flow cytometry

For each condition, 10^6^ cells were washed in PBS 1X at 4°C. Fluorescence was analyzed on a FACScan cytometer (BD Biosciences^®^), and the data were analyzed with CellQuest (BD Biosciences^®^). The measurements were performed twice in two independent experiments.

### Immunohistochemistry

Immunohistochemistry used standard procedures. Briefly, tumors were fixed in 4% paraformaldehyde and embedded in paraffin. Sections (4 μm) were deparaffinized and heated for 10 minutes in 10 mmol/L citrate buffer, pH 6.2, for antigen retrieval. They were stained with Harris solution and Eosin for histological examination and immunostained using the primary antibodies raised against CXCR4 (1:200; eBiosciences) and CXCR7 (1:75; ThermoScientific). Slides were then incubated for 30 min with secondary biotinylated anti-mouse antibody (dilution 1:200; Vector Laboratories Inc., Burlingame, CA). Immunostaining was developed with a liquid DAB substrate kit (Roche Diagnostics) according to the manufacturer’s instructions.

### Statistics

The data were analyzed with the Mann–Whitney parametric test, and the significance level was set at 5%.

## Competing interests

The authors declare they have no competing interests or other interests that might be perceived to influence the results and discussion reported in this paper.

## Authors’ contributions

BR and MHH performed all experiments. BR, SR and CB contributed to the surgical specimen, patient consent and clinical data collection. JLG and MPG revised the paper critically for important intellectual content. EP and DG performed the conception and design of the study as well as the final approval of the version to be published. All authors have read and approved the final manuscript.
